# Hydrogen Activation via Dihydride Formation on a Rh_1_/Fe_3_O_4_(001) Single‐Atom Catalyst

**DOI:** 10.1002/anie.202525745

**Published:** 2026-02-18

**Authors:** Chunlei Wang, Panukorn Sombut, Lena Puntscher, Nail Barama, Maosheng Hao, Florian Kraushofer, Jiri Pavelec, Matthias Meier, Florian Libisch, Michael Schmid, Ulrike Diebold, Cesare Franchini, Gareth S. Parkinson

**Affiliations:** ^1^ Institute of Applied Physics TU Wien Vienna Austria; ^2^ Institute of Theoretical Physics TU Wien Vienna Austria; ^3^ Faculty of Physics Center For Computational Materials Science University of Vienna Vienna Austria; ^4^ Dipartimento Di Fisica e Astronomia Università Di Bologna Bologna Italy

**Keywords:** density functional theory, metal‐oxide surfaces, scanning tunneling microscopy, single‐atom catalysis, hydrogen adsorption

## Abstract

Hydrogen activation is a key elementary step in catalytic hydrogenation. In heterogeneous catalysis, it usually proceeds through dissociative adsorption on metal nanoparticles followed by surface diffusion or spillover, whereas homogeneous catalysts activate H_2_ through dihydride or dihydrogen intermediates at a single metal center. Here, we show that isolated Rh adatoms supported on Fe_3_O_4_(001) activate hydrogen through formation of a stable dihydride species without atomic H spillover. Temperature‐programmed desorption, x‐ray photoelectron spectroscopy, and scanning tunneling microscopy collectively reveal strong (≈1 eV) hydrogen adsorption exclusively at isolated Rh_1_ sites, while isotope‐exchange experiments further demonstrate that hydrogen remains localized. Density‐functional theory‐based calculations indicate a barrierless conversion from molecular H_2_ to the dihydride, and random‐phase approximation calculations further confirm the relative stability of the dihydride. Together, these results show that single‐atom Rh sites cleave hydrogen through a dihydride pathway analogous to homogeneous complexes, establishing a mechanistic bridge between homogeneous and heterogeneous catalysis.

## Graphical abstract

Deuterium (hydrogen) adsorption on single‐atom Rh_1_/Fe_3_O_4_(001) catalyst does not induce D (H) spillover onto the reducible Fe_3_O_4_ support. Instead, a Rh_1_‐dihydride (Rh−2D or Rh−2H) species forms and D_2_ or H_2_desorbs during annealing.

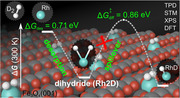

## 1 Introduction

Catalysis plays a critical role in the modern economy, and tremendous effort is devoted to improving catalytic performance in terms of activity, selectivity, and atomic efficiency [1, 2]. Hydrogenation, the process of adding hydrogen to unsaturated molecules, is a key transformation used in fine chemical synthesis, petroleum refining, and pharmaceutical production [3]. Understanding how catalysts activate H_2_ is therefore of fundamental importance, as this step often determines both rate and selectivity [4, 5, 6].

Hydrogenation can be achieved by both heterogeneous and homogeneous catalysts. Industrial processes typically prefer heterogeneous systems because they are robust, easy to separate from products, and reusable. Homogeneous catalysts, by contrast, excel when high selectivity, mild conditions, and precise control over molecular transformations are required [7]. Bridging the advantages of both regimes, by combining molecular precision with practical operability, has long been a central goal in catalysis research [8, 9, 10].

In homogeneous catalysis, the metal center is coordinated by molecular ligands, and H_2_ activation is generally achieved by formation of a dihydride or dihydrogen complex in which the H─H bond is cleaved or elongated [11, 12, 13]. These intermediates are widely regarded as the key to the high reactivity and selectivity observed in homogeneous catalysis. In contrast, hydrogen activation on supported metal catalysts usually occurs through dissociative adsorption on nanoparticles, followed by migration of atomic hydrogen either across the metal surface or onto the support (hydrogen spillover) [14, 15]. Although such mechanisms can enhance overall activity, the resulting mobile hydrogen reservoir often compromises selectivity for partially hydrogenated products [7]. This highlights the need for alternative activation modes on solid catalysts that could emulate the controlled reactivity of molecular systems.

Single‐atom catalysts (SACs), in which individual metal atoms are anchored to a support, offer such an alternative [16]. These are heterogeneous catalysts but possess structural similarities to homogeneous systems, particularly in their metal‐ligand coordination environment. As a result, SACs have been proposed as a platform to bridge homogeneous and heterogeneous catalysis [1, 10, 17, 18]. However, beyond structural resemblance, achieving this bridge requires that SACs operate through similar catalytic mechanisms. H_2_ activation on SACs is commonly reported to proceed via metal–H formation or through hydrogen spillover onto the supports [19, 20, 21, 22, 23]. For example, Doudin et al. observed hydrogen dissociation and spillover on a Pd_1_/Fe_3_O_4_(001) model SAC at room temperature [24]. More recently, Pacchioni and coworkers calculated that stable dihydride and dihydrogen complexes could also form on SACs, with important implications for the electrochemical hydrogen evolution reaction [25]. However, direct experimental evidence for such species under well‐defined conditions has remained elusive.

In this work, we experimentally demonstrate that Rh_1_ species on a reducible Fe_3_O_4_(001) support strongly adsorb H_2_ without atomic hydrogen migration and spillover. Instead, a dihydride species forms, analogous to H_2_ activation in homogeneous catalysis. These findings suggest that heterogeneous SACs can provide mechanisms similar to those of highly selective homogeneous catalysts.

## 2 Results and Discussion

**FIGURE 1:**
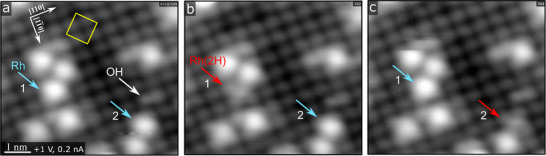
Time‐lapse STM shows reversible H_2_ adsorption and desorption on 0.2 ML Rh/Fe_3_O_4_(001). STM was conducted at room temperature under a partial pressure of 4 × 10^−9^ mbar H_2_. The three frames of STM images depict representative states of Rh adatoms during H_2_ exposure. Sites that adsorb H_2_ exhibit reduced apparent height (red arrows in b and c). The cyan arrows may represent the Rh_1_ states either prior to H_2_ adsorption or following H_2_ desorption. Some surface hydroxyls were already formed on Fe_3_O_4_(001) during the initial sample preparation (marked with a white arrow). The yellow square in (a) marks the reconstructed surface unit cell of Fe_3_O_4_(001).

The Fe_3_O_4_(001) surface reconstructs into a (√2 × √2)R45° pattern when prepared under UHV (Figure 1a, yellow square). This structure arises from an ordered arrangement of subsurface cation vacancies and interstitials, resulting in an oxidized surface layer [26]. The as‐prepared surface contains several defects, most notably hydroxyl species marked by white arrows in Figures 1a and S1a. These originate from dissociative adsorption of trace water at oxygen vacancies created during sputtering and annealing. Although hydroxyls are not directly visible in STM, they modify the contrast of nearby Fe atoms, which appear brighter in empty‐state images [27, 28]. This assignment has been confirmed by direct deposition of atomic hydrogen [29]. At room temperature, the hydroxyls hop between two equivalent oxygen sites within a surface unit cell, producing a characteristic back‐and‐forth motion easily recognized in STM movies [27]. Apart from the hopping, the number and position of the hydroxyls remain unchanged throughout the following measurements.

For STM measurements, 0.2 monolayers (ML) of Rh were deposited onto the as‐prepared Fe_3_O_4_(001) surface at room temperature. Individual Rh adatoms are marked by cyan arrows in Figure 1. They appear as bright protrusions located midway between surface Fe rows, with a nearest‐neighbor distance of 8.34 Å. The Rh adatoms bind to two surface oxygen atoms on opposite rows (see Figure S1(b–d), consistent with the behavior of other metals on this surface) [30, 31, 32]. Additionally, the 2‐fold Rh─O coordination observed on Fe_3_O_4_(001) is stable under reactive environments [30, 33], including high‐pressure CO exposure [34]. Time‐lapse STM in 4 × 10^−9^ mbar H_2_ reveals reversible height changes at individual Rh_1_ sites (Figure 1b,c). The apparent height of a Rh_1_ adatom is typically 140–160 pm but transiently decreases to 40–70 pm during H_2_ adsorption. These fluctuations occur randomly and infrequently, indicating reversible H_2_ adsorption at room temperature. No new bright features or hydroxyls appear near the affected sites, confirming that hydrogen remains localized at Rh_1_ and does not spill over onto the oxide lattice. An STM movie covering a longer time span and larger area than Figure 1 is provided in the supporting information (Movie S1).

**FIGURE 2:**
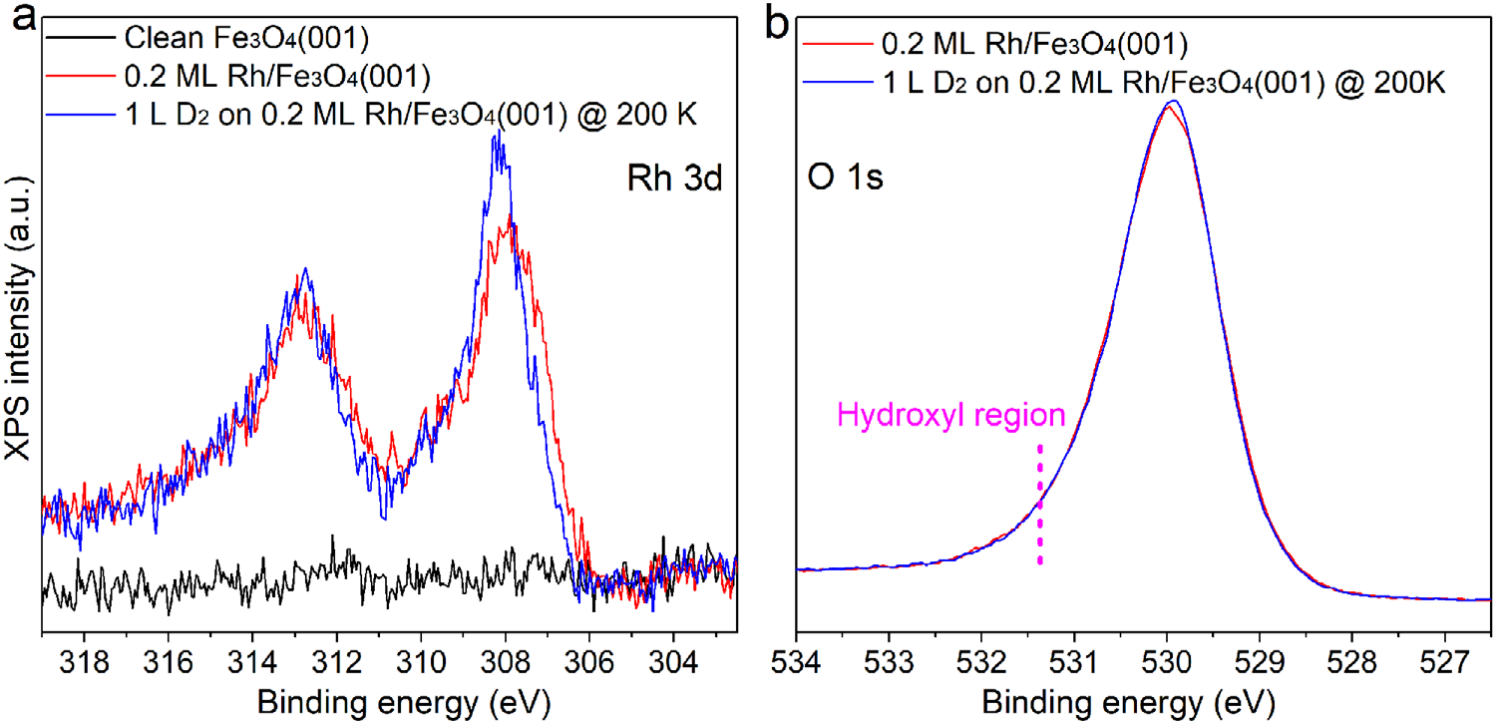
XPS confirms lack of hydroxyl formation. XPS spectra of the (a) Rh 3d and (b) O 1s regions collected from clean Fe_3_O_4_(001) and from 0.2 ML Rh on Fe_3_O_4_(001) before and after 1 Langmuir (L) D_2_ exposure at 200 K. 1 L is defined as 1.33 × 10^−^^6^ mbar·s. The pink dashed line in (b) marks the OD region in the O 1s spectrum.

XPS spectra of the Rh 3d and O 1s regions were recorded for 0.2 ML Rh/Fe_3_O_4_(001) before and after D_2_ exposure. After adsorption at 200 K, the Rh 3d peak sharpens and shifts slightly to higher binding energy (Figure 2a), indicating modification of the Rh_1_ electronic state. In the O 1s region, no new feature appears near 531.3 eV where hydroxyl species are expected on Fe_3_O_4_(001) [35, 36], even after prolonged D_2_ exposure (10 L at 250 K, Figure S2). The absence of this signal confirms that hydrogen remains bound to Rh_1_ and does not migrate onto the oxide lattice.

**FIGURE 3:**
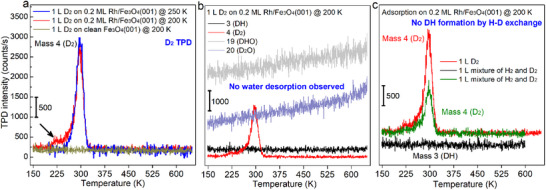
TPD and isotope exchange experiments. (a) TPD spectra acquired after exposing 0.2 ML Rh/Fe_3_O_4_(001) to 1 L D_2_ at 250 and 200 K. A reference spectrum was also recorded after exposing clean Fe_3_O_4_(001) to 1 L D_2_ at 200 K. Only the mass 4 (D_2_) signal is shown. The small arrow indicated stems from the sample holder (see Figures S4 and S5). (b) Representative TPD data collected after 1 L D_2_ exposure on 0.2 ML Rh/Fe_3_O_4_(001) at 200 K, showing signals for mass 3 (DH), 4 (D_2_), 19 (DHO), and 20 (D_2_O). (c) TPD after dosing pure D_2_ and an equimolar 1 L mixture of D_2_ and H_2_ (1:1 partial pressure ratio) at 200 K. No isotope scrambling was observed. In all TPD experiments, the sample was cooled by 50 K after dosing to achieve a perfectly linear temperature ramp in the region where desorption occurs. Curves in (c) are vertically offset for clarity.

To quantify the strength of hydrogen adsorption on Rh/Fe_3_O_4_(001), TPD experiments were performed using D_2_ to avoid interference from residual H_2_ in the vacuum system. The clean Fe_3_O_4_(001) surface shows no detectable D_2_ desorption after 1 L exposure at 200 K (Figure 3a, olive trace), confirming that molecular hydrogen binds only weakly to the oxide as on other oxide surfaces [37]. After deposition of 0.2 ML Rh_1_ and exposure to 1 L D_2_ at 250 K, a distinct D_2_ desorption peak appears at 295 K (Figure 3a, blue trace), consistent with the transient adsorption observed by STM (Figure 1).

Lowering the dosing temperature to 200 K (Figure 3a, red trace) or 150 K (Figure S3, pink trace) does not reveal additional adsorption states. A weak shoulder near 225 K is identified as an artifact from D_2_ adsorption on the Ta sample holder (Figures S4 and S5). Analysis of the main desorption peak using a recently introduced method based on equilibrium thermodynamics [38] (Figure S6) yields an adsorption energy of 0.96 ± 0.06 eV, indicating strong but reversible binding of hydrogen to Rh_1_ sites.

No D_2_O desorption is detected up to 600 K (Figure 3b), ruling out formation of OD species on the support (recombinative water desorption occurs at ≈550 K on Fe_3_O_4_(001)) [24]. Even at higher doses (5–10 L) or elevated exposure temperatures (200–295 K), no spillover products appear (Figures S7 and S8). Signals corresponding to DH or DHO are also absent, excluding isotope scrambling or recombination with residual H_2_.

Exposure of Rh_1_/Fe_3_O_4_(001) to an equimolar H_2_/D_2_ mixture provides a further test for hydrogen mobility. The D_2_ signal decreases by roughly half relative to pure D_2_ dosing (Figure 3c), as expected for competitive adsorption with H_2_, but no DH signal is observed. Thus, hydrogen adsorbs and desorbs exclusively at isolated Rh_1_ sites without spillover or isotope exchange. The H_2_ signal is not shown due to the comparably high background in UHV chamber, hiding the H_2_ peak in the noise, see Figure S9 for details.

Although individual Rh adatoms seemingly appear in close proximity in STM images, the minimum Rh–Rh separation on Fe_3_O_4_(001) is sufficiently large to preclude direct Rh–Rh bonding. Importantly, hydrogen adsorption and desorption are characterized by a single, well‐defined state in TPD and isotope‐exchange experiments, indicating that H_2_ activation occurs at a unique Rh_1_ site. No additional hydrogen‐related features attributable to Rh–Rh ensembles are observed, demonstrating that the dihydride species identified here is intrinsic to isolated Rh atoms rather than to clustered sites.

**FIGURE 4:**
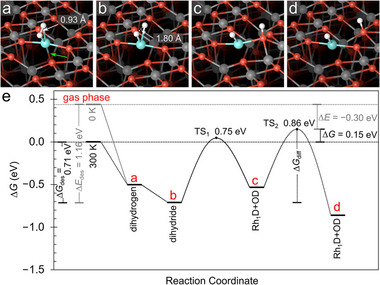
DFT‐calculated mechanism of D_2_ dissociation and spillover on Rh_1_/Fe_3_O_4_(001). Perspective views are shown for (a) a dihydrogen configuration and (b) a dihydride configuration. In both cases, the Rh_1_ species is bonded to two surface oxygen atoms. An additional weak bond from the Rh to a subsurface O atom (roughly 0.3 Å shorter than in the Rh2D configuration) is indicated by the green arrow. (c and d) Atomic structures of atomic D spillover along the Fe_3_O_4_(001) surface. Fe atoms are shown in dark gray, and oxygen atoms are red. (e) Computed reaction energy profile for molecular D_2_ adsorption, dissociation, and hydrogen spillover from a Rh_1_ site onto the Fe_3_O_4_(001) substrate. The reported Gibbs free energy (Δ*G*) values incorporate the entropy difference between the adsorbed state (Assuming *S_ads_* =  0) and the gas phase (*S*_gas_) (as referenced in database) [39]. These Δ*G* values are presented for the experimental desorption temperature (300 K) and derived from DFT calculations that include ZPE corrections. The overall reaction mechanism remains identical to the 0 K pathway, except the desorption step, for which the 0 K desorption energy (Δ*E_ads_*) is provided for comparative reference. Transition states (TS_1_ and TS_2_) and the corresponding activation barriers with respect to the dihydride state (b) are indicated.

Density functional theory (DFT+*U*) calculations were performed to clarify the nature of deuterium adsorption on Rh_1_/Fe_3_O_4_(001). In the following, all values include zero point energy (ZPE) corrections for D_2_, with corresponding results for H_2_ shown in the Supporting Information. Two local minima were identified (Figure 4a,b). The weak‐binding configuration is a dihydrogen‐like complex with the D─D distance of 0.93 Å and an adsorption energy of −0.94 eV. In this case, the Rh atom relaxes slightly toward a subsurface oxygen (green arrow in Figure 4a), forming a pseudo‐square‐planar geometry similar to that found for CO and C_2_H_4_ adsorption on Rh_1_/Fe_3_O_4_(001) [33, 40].

The second, more strongly bound structure corresponds to a dihydride configuration, where the D─D distance is 1.8 Å and the adsorption energy is −1.16 eV. The two deuterium atoms occupy positions above the Rh center, giving the metal a tetrahedral coordination geometry (Figure 4b). This contrasts with the square‐planar configuration observed previously for gem‐dicarbonyl species on the same surface [40]. This is consistent with the classical ligand‐field expectations: weak‐field hydride ligands favor tetrahedral coordination, whereas strong‐field CO ligands stabilize a square‐planar arrangement [41].

To address the sensitivity of the DFT+U calculations to self‐interaction errors, we benchmarked hydrogen binding energetics using the r^2^SCAN + U and HSE06 functionals, as well as the random phase approximation (RPA) method. The identical energetic ordering obtained across all methods (see Table S1) corroborates the robustness of our results. The dihydride state is lower in energy, and CI‐NEB calculations suggest a barrierless conversion from the molecularly adsorbed D_2_ (dihydrogen) precursor to the dihydride product. This transition closely parallels activation mechanisms proposed for homogeneous transition‐metal complexes [11]. In principle, advanced infrared spectroscopy on SACs can provide direct experimental characterization of hydrogen complexes through M–H vibrational modes, as has been reported [42, 43]. However, in our case, the expected IR signals are severely limited by the low loading of metal single atoms and the weak IR activity of hydrogen species [44].

DFT + U calculations suggest that two D_2_ molecules could, in principle, adsorb on a single Rh_1_ atom, with a combined adsorption energy of −1.62 eV (−0.81 eV per D_2_; Figure S10). Experimentally, however, only one desorption state is observed, and H–D exchange experiments show no isotope scrambling. The absence of a second adsorption state indicates kinetic hindrance, as accommodating a second molecule would require substantial distortion of the Rh center. Similar geometric constraints were previously found for CO adsorption, where binding an additional ligand necessitates vertical displacement of Rh and lateral rearrangement of the first adsorbate. Due to the energetic cost, this is a rare process that is unlikely to coincide with adsorption events under UHV conditions [40].

We have also considered hydrogen adsorption on substitutional (5‐fold coordinated) Rh atoms, which can be also present in this surface (especially after annealing to higher temperatures than used here; see also Figure S1b). DFT + U shows that hydrogen adsorption is weak (−0.39 eV and −0.44 eV for H_2_ and D_2_, respectively) and dissociation (dihydride formation) does not occur at these sites. This agrees with the notion that dihydride formation requires undercoordinated metal atoms [25].

To understand the lack of spillover at room temperature in the experiments, entropic contributions to the free energy Δ*G* must be taken into account. Figure 4e compares the free‐energy profiles for hydrogen desorption and for hydrogen migration from Rh to a neighboring surface oxygen. Spillover would proceed via an initial hydrogen transfer from Rh_1_ to a neighboring oxygen atom, followed by diffusion away from the metal center (Figure 4c,d).

The calculated activation barrier for hydrogen migration from Rh_1_ to a neighboring surface oxygen and subsequent diffusion away from the metal center is 0.86 eV (Figure 4c,d). In contrast, when entropic contributions at 300 K are included, the free‐energy barrier for recombinative D_2_ desorption is 0.71 eV (Figure 4e). Because the spillover barrier exceeds the desorption barrier, hydrogen desorption is kinetically preferred under the experimental conditions, explaining the absence of spillover in experiment.

At first glance, the absence of hydrogen spillover may seem counterintuitive given that the final spillover state is thermodynamically accessible, and more favorable than desorption when disregarding entropy. However, spillover and desorption are fundamentally different processes. Spillover requires a localized, rearrangement at the Rh–O interface. Compared to desorption, it is entropically disfavored because both hydrogen atoms remain surface bound. In contrast, desorption releases H_2_ into the gas phase, where the large translational entropy strongly stabilizes the transition state. Kinetics are governed by free‐energy barriers rather than the mere DFT‐calculated energy barriers. Until desorption, this effectively confines hydrogen to the Rh_1_ site under the conditions studied here. The corresponding free‐energy profile for H_2_ is provided in Figure S11.

The Δ*G* given in Figure 4e adopt the commonly used approximation that the entropy contribution of adsorbed species is negligible (*S*_ads_ ≈ 0), which is generally considered reasonable for small adsorbates such as hydrogen [45]. In our case, the relevant entropy contributions in the adsorbed state are (i) vibrations of the adsorbed deuterium and (ii) the change of the Rh vibrations associated to its transition from a 2‐fold geometry to that in the dihydride. The vibration frequencies are given in Table S2. The effect of the deuterium vibrations on adsorption is small (disfavoring desorption by 0.024 eV at 300 K; less for hydrogen). The entropy of the Rh_1_ adatom slightly decreases upon adsorption (increased vibration frequencies), making desorption more favorable by 0.010 eV. These effects are much smaller than typical DFT errors. Due to the low mass (high vibration frequencies) of H and D, we can also neglect entropy changes upon diffusion to the oxide, which would modify the energy landscape of the spillover process. What remains is the large entropy contribution of the gas phase, which strongly favors desorption in spite of an overall energy gain in the final state of spillover (Figure 4e).

One particularly revealing aspect of the computed energy landscape is the presence of a local minimum in which one deuterium atom transfers to the oxygen atom directly bonded to Rh (Figure 4c). This configuration lies 0.18 eV above the dihydride minimum, corresponding to an equilibrium population of ≈ 1 × 10^−3^ at 300 K. Although too sparse to be detected by STM, XPS, or TPD, such thermally accessible excitations imply that transient heterolytic D cleavage can occur within the Rh–O ensemble. The calculated activation barrier for this intrapair D transfer (0.75 eV) is slightly lower than that for full spillover (0.86 eV), suggesting that deuterium can shuttle locally between Rh and the neighboring oxygen without diffusing away. Importantly, this transient hydrogen shuttling remains confined to the immediate Rh─O coordination sphere and does not constitute hydrogen spillover in the classical sense. Rather than diffusive migration across the oxide surface, this local metal–support cooperation temporarily frees a coordination site on Rh, enabling adsorption and reaction of unsaturated molecules directly at the Rh_1_ site. Hydrogenation can therefore proceed via localized hydrogen transfer from the dihydride without requiring long‐range hydrogen transport. This process therefore constitutes metal–ligand‐type cooperation confined to a single site, rather than hydrogen (deuterium) spillover in the classical sense [14], providing a clear mechanistic distinction between local heterolytic activation and long‐range D migration across the surface.

The resulting “split‐hydride” configuration closely parallels bifunctional H_2_ activation in molecular complexes, where the H–H bond is cleaved heterolytically across a metal–ligand pair [11, 12, 13]. In this context, the Rh–O pair acts as a functional analogue of a metal–ligand site capable of transient H storage and retrieval. This behaviour represents a key step toward replicating the controlled, site‐specific reactivity characteristic of homogeneous hydrogenation catalysts.

More broadly, the combined experimental and theoretical results demonstrate that isolated Rh atoms on Fe_3_O_4_(001) strongly bind and activate H_2_ without forming a mobile hydrogen reservoir. The stable dihydride configuration identified here experimentally agrees with recent theoretical predictions that SACs can host discrete M–H_2_ or metal‐dihydride species [25]. By confining hydrogen to well‐defined metal‐hydride species, the system suppresses spillover and prevents the excessive hydrogen availability that typically causes over‐hydrogenation on nanoparticle catalysts. This localization is particularly relevant for selective hydrogenation reactions such as the conversion of acetylene to ethylene, where controlled hydrogen delivery is essential for maintaining high selectivity [46, 47]. Comparable control has been reported in other single‐atom hydrogenation catalysts [18, 19], supporting the view that geometric confinement and the covalent metal–oxide interaction stabilize molecular‐like hydride species while retaining the robustness of a solid surface. These findings thus provide direct mechanistic evidence that SACs can bridge the conceptual gap between homogeneous and heterogeneous hydrogenation.

## 3 Conclusions

Our combined experimental and theoretical analysis demonstrates that isolated Rh atoms on Fe_3_O_4_(001) activate hydrogen through the formation of kinetically stabilized dihydride species, without hydrogen spillover to the support. This behavior mirrors the H_2_ activation mechanism of homogeneous catalysts, establishing direct mechanistic correspondence between molecular and supported single‐site systems. The findings demonstrate that isolated single atoms can host discrete metal‐hydride species at finite temperature, which is required for enabling selective hydrogenation pathways inaccessible to extended metal surfaces. By translating homogeneous dihydride formation to a solid platform, this work provides a foundation for designing SACs that unite molecular‐level precision with the robustness and scalability of heterogeneous catalysis.

## Conflicts of Interest

The authors declare no conflicts of interest.

## Supporting information




**Supporting File 1**: anie71466‐sup‐0001‐SuppMat.pdf.

**Supporting File 2**: anie71466‐sup‐0002‐MovieS1.avi.

## Data Availability

The data that support the findings of this study are available from the corresponding author upon reasonable request.
