# Supplementary material for: Hydrogen Activation via Dihydride Formation on a Rh1/Fe3O4(001) Single‐Atom Catalyst
Source: Angew Chem Int Ed Engl. 2026 Feb 18;65(14):e25745. doi: 10.1002/anie.202525745 (PMC13023702; doi:10.1002/anie.202525745)
Supplement: Supplementary file 1 — Supporting File 1: anie71466‐sup‐0001‐SuppMat.pdf. [file ANIE-65-e25745-s001.pdf]

Supplementary Information:

Hydrogen Activation via Dihydride Formation on a $Rh_1/Fe_3O_4(001)$ Single-Atom Catalyst

Chunlei Wang[1], Panukorn Sombut[1], Lena Puntscher[1], Nail Barama[1], Maosheng Hao[2], Florian Kraushofer[1], Jiri Pavelec[1], Matthias Meier[1,3], Florian Libisch[2], Michael Schmid[1], Ulrike Diebold[1], Cesare Franchini[3,4], and Gareth S. Parkinson[1]

[1]Institute of Applied Physics, TU Wien, Vienna, Austria

[2]Institute of Theoretical Physics, TU Wien, Vienna, Austria

[3]Faculty of Physics, Center for Computational Materials Science, University of Vienna, Vienna, Austria

[4]Dipartimento di Fisica e Astronomia, Università di Bologna, Bologna, Italy

The supplementary material includes detailed descriptions of both experimental and computational methods, as well as additional results:

1) Additional STM Data
2) Additional XPS data
3) Additional TPD data
   a. Origin of shoulder in D_2 TPD traces
   b. Deriving desorption energies from TPD
   c. Variation of D_2 dosages and adsorption temperatures
   d. H−D isotope exchange experiments
4) Additional computational results
   a. Comparison of adsorption energies with various computational approximations
   b. Higher H_2 loading
   c. Estimate of kinetics for hydrogen desorption vs spillover

Other Supplementary Material for this manuscript includes the following:

Movie S1: Time-lapse STM of 0.2 ML $Rh/Fe_3O_4(001)$ surface under exposure to 4×10^{-9} mbar H_2 at room temperature.

Experimental and Computational Methods

Experimental

Experiments were performed on natural $Fe_3O_4(001)$ (6×6×1 mm) single crystals purchased from SurfaceNet GmbH. Two separate ultra-high vacuum (UHV) systems were used for scanning tunneling microscopy (STM) imaging and for temperature-programmed desorption (TPD)/X-ray photoelectron spectroscopy (XPS) experiments, respectively. Samples were cleaned by repeated cycles of sputtering and annealing in UHV. Sputtering was performed using 1 keV Ar^+ ions (STM chamber) or Ne^+ ions (TPD/XPS chamber) for 10 minutes, followed by annealing at 900 K for 10 minutes. Annealing in oxygen was used to reoxidize the surface after several cleaning cycles. A final annealing step in 5×10^{-7} mbar O_2 at 900 K results in the formation of the reconstructed ($\sqrt{2} \times \sqrt{2}$)R45°surface.[1] Rh atoms were deposited using an electron-beam evaporator (FOCUS), and the flux was calibrated with a temperature-stabilized quartz microbalance. The coverage of one monolayer (ML) is defined as one Rh atom per $Fe_3O_4(001)$-($\sqrt{2}\times\sqrt{2}$)R45° surface unit cell (area density of 1.42×10^{14} cm^{-2}).

In the TPD chamber, the $Fe_3O_4(001)$ sample was mounted on a Ta backplate, with a thin gold sheet inserted in between to improve thermal contact. The sample was cooled using a liquid-He flow cryostat and heated resistively via the Ta backplate. The chamber includes a home-built molecular beam source that delivers reactants with a calibrated flux (equivalent to an impingement rate of a room-temperature gas at a pressure of 2.66×10^{-8} mbar) and a uniform top-hat profile across a 3.5 mm diameter spot on the sample.[2] Gas exposure is given in Langmuirs (L), where 1 L is defined as 1.33×10^{-6} mbar·s. A quadrupole mass spectrometer (Hiden HAL 3F PIC) was used for TPD experiments. Deuterium (D_2) was employed instead of H_2 to avoid background interference from residual H_2 in the vacuum chamber. TPD spectra were acquired using a temperature ramp of 1 K/s. The chamber was also equipped with a monochromatic Al/Ag twin-anode X-ray source (Specs XR50 M, FOCUS 500) and a hemispherical analyzer (Specs Phoibos 150) for XPS measurements. A grazing angle of $\approx$71° with respect to surface normal was used to collect the XPS spectra.

STM studies were conducted using an Omicron μ-STM operated in constant-current mode at room temperature, with an electrochemically etched W tip. A positive sample bias was used in all cases, enabling measurement of the unoccupied electronic states. Sample preparation and Rh deposition were performed in an adjacent chamber. H_2 was introduced directly into the analysis chamber for STM time-lapse imaging experiments. The analysis chamber was also equipped with a non-monochromated Al Kα X-ray source and a SPECS Phoibos 100 analyzer for XPS measurements.

Computational Details

All calculations were performed using the Vienna *ab initio* simulation package (VASP).[3] The projector augmented-wave (PAW) method[4] was used to describe the near-core regions, and a plane-

wave energy cutoff of 550 eV was used. The generalized gradient approximation (GGA) in the form of the Perdew-Burke-Ernzerhof (PBE) functional[5] was used to treat electronic exchange and correlation effects. Dispersion corrections were included following the D3 scheme with Becke-Johnson damping.[6] To adequately describe the strongly correlated Fe 3d electrons, an effective on-side Coulomb interaction U_{eff} of 3.61 eV was applied.[7] The electronic energy convergence criterion was set to 10^{-6} eV, and ionic relaxation was performed until the forces acting on ions became smaller than 0.02 eV/Å. The magnetite (Fe_3O_4) system was modelled using the experimentally reported lattice parameter of a = 8.396 Å. The asymmetric slab consisted of 7 octahedral Fe and 6 tetrahedral Fe layers. During relaxation, only the topmost 4 layers were allowed to relax, while the bottom 9 layers remained fixed at their bulk positions. Calculations employed a $(2\sqrt{2} \times 2\sqrt{2})R45^\circ$ surface supercell, and Brillouin zone sampling was restricted to the Γ-point only due to the large cell size. A vacuum spacing of 14 Å was included to prevent interaction between periodic images. To account for the small mass of H, zero-point energy (ZPE) corrections were explicitly included in adsorption energy calculations. The adsorption energy (ΔE_{ads}) was computed according to the formula:

$$\Delta E_{ads} = \left(E_{\mathrm{Rh/Fe_3O_4}+n\mathrm{H_2}} - \left(E_{\mathrm{Rh/Fe_3O_4}} + nE_{\mathrm{H_2}}\right)\right) + \Delta E_{\mathrm{ZPE}}$$

where $E_{\mathrm{Rh/Fe_3O_4}+n\mathrm{H_2}}$ represents the total energy of the Rh-adatom-decorated Fe_3O_4(001) surface with n adsorbed H_2 molecules, $E_{\mathrm{Rh/Fe_3O_4}}$ denotes the total energy of the Rh-decorated surface without any adsorbates, and $E_{\mathrm{H_2}}$ corresponds to the energy of an isolated H_2 molecule in the gas phase. The zero-point energy correction (ΔE_{ZPE}) was obtained by calculating vibrational frequencies using the finite-difference method. Only the adsorbed hydrogen atoms, the Rh center, and the oxygen atoms bonded to the hydrogen along the proposed diffusion pathway were allowed to be displaced, while all the other atoms were kept fixed at their optimized positions.

The Gibbs free energy at T = 300 K was then calculated as:

$$\Delta G_{ads} = \Delta E_{ads} - T\Delta S$$

where ΔS is the entropy change between the adsorbed species (taken as S = 0) and the gas phase.[8] Diffusion activation energies were calculated using the climbing-image nudged elastic band (CI-NEB)[9] method. The electronic self-consistency criterion was set to 10^{-6} eV, and the forces on atoms in each image were relaxed to below 0.05 eV/Å.

Additional calculations were performed using the regularized-restored strongly constrained and appropriately normed meta-generalized gradient approximation (r^2SCAN) [10] with the U_{eff} of 3.10 eV[11] and the hybrid functional HSE06[12] to validate results and assess the accuracy of the employed methods. To further assess the effects of electronic correlation, we also performed additional random-phase approximation (RPA) calculations based on the hybrid DFT orbitals, using the more accurate PAW projectors provided with VASP to better capture the local electronic structure around the Rh atom.[13]

Due to computational limitations associated with the post-DFT methods, calculations using HSE06[12] and RPA were conducted with a smaller $(\sqrt{2} \times \sqrt{2})R45°$ cell, still restricting Brillouin-zone sampling to the Γ-point. The finite-size effect was tested using DFT+U. For the $2H/Rh_1$ (dihydride) configuration, the difference in adsorption energy between the two cell sizes was approximately 0.06 eV at the PBE+D3(BJ) level.

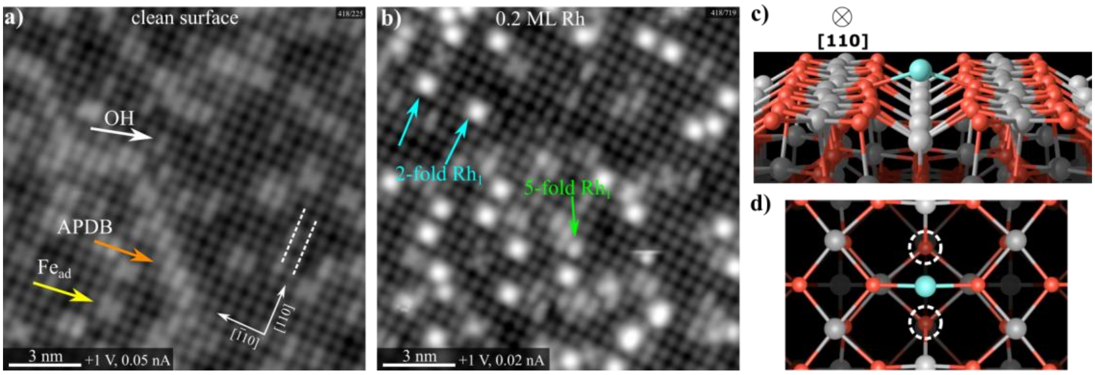

Figure S1. Rh/Fe_3O_4(001) model catalyst structure. (a) STM image of the clean Fe_3O_4(001) surface exhibiting the (√2×√2)R45° reconstruction. The bright protrusions correspond to pairs of surface Fe atoms oriented along the [110] direction, thus forming Fe rows in this direction (white dashed lines).[1] Oxygen atoms are not visible in empty-state STM due to their low density of states near the Fermi level. Surface defects, including hydroxyl groups (OH), antiphase domain boundaries (APDB), and Fe adatoms (Fe_{ad}), are marked with white, orange, and yellow arrows, respectively. These defects have been characterized previously.[14] (b) STM image after deposition of 0.2 ML Rh at 300 K. Twofold oxygen-coordinated Rh adatoms appear between Fe rows (cyan arrows). Less common fivefold coordinated Rh adatoms are also observed (green arrow), as reported earlier.[15] (c, d) Perspective and top-view DFT-optimized structures of a twofold coordinated Rh_1 atom on the reconstructed Fe_3O_4(001) surface. The dashed circles in (d) indicate two equivalent subsurface oxygen atoms that can weakly interact with Rh_1 upon molecular adsorption.

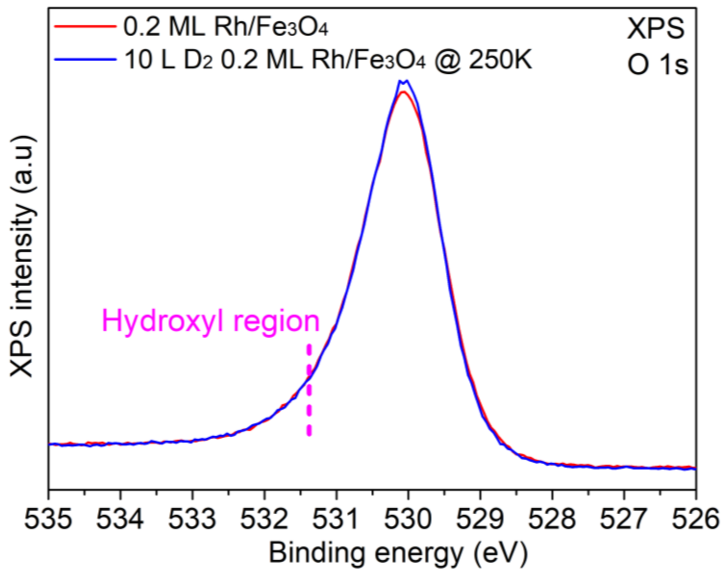

Figure S2. XPS analysis confirms absence of OD formation after extended D_2 exposure. O 1s XPS of 0.2 ML Rh/Fe_3O_4(001) before and after exposure to 10 L D_2 at 250 K. The XPS was collected with a grazing angle of ≈ 71°. The pink dashed line marks the energy of OH (or OD) signals.[16] Even under these thermodynamically and kinetically favorable conditions (compared to 1 L D_2 at 200 K in Figure 2b), no OD-related signal is observed. This confirms that atomic hydrogen spillover onto the Fe_3O_4 support does not occur in this Rh single-atom catalyst system.

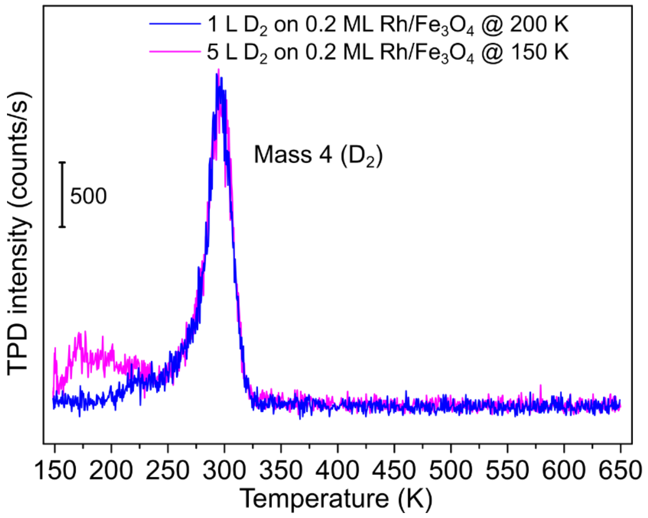

Figure S3. Saturation behavior of D_2 adsorption on Rh/Fe_3O_4(001). TPD spectra following adsorption of 1 L D_2 at 200 K (blue) and 5 L D_2 at 150 K on 0.2 ML Rh/Fe_3O_4(001). Despite differences in exposure temperature and dose, both spectra exhibit an overlapping desorption peak centered around 295 K, indicating that 1 L D_2 at 200 K is sufficient to saturate the Rh sites. The shoulder at 150–220 K is attributed to D_2 adsorption on Rh deposited onto the Ta sample plate. This contribution is further examined in the following figures.

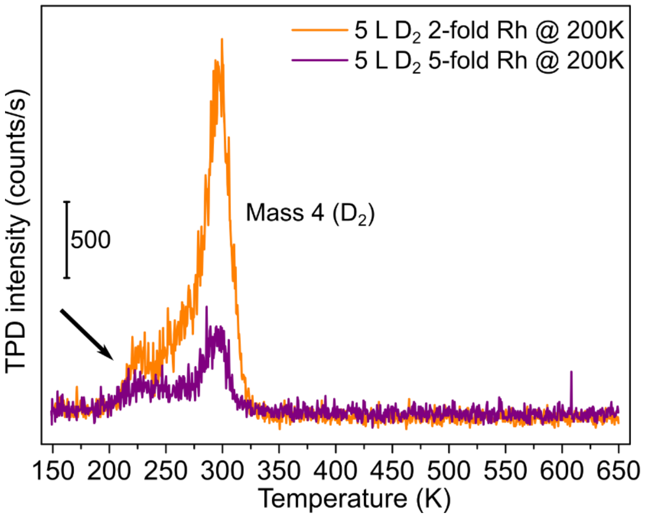

Figure S4. Control experiment to assess the contribution of 5-fold coordinated Rh to D_2 desorption. TPD spectra recorded after 5 L D_2 adsorption at 200 K on two Rh/Fe_3O_4(001) samples: one dominated by twofold coordinated Rh adatoms (orange) and another with a higher density of fivefold coordinated Rh adatoms embedded in the surface (purple).[15b] Both spectra exhibit a low-temperature shoulder (black arrow), but the signal intensity does not increase in the sample containing more fivefold Rh sites. This confirms that the shoulder feature does not originate from D_2 adsorption at fivefold Rh adatoms.

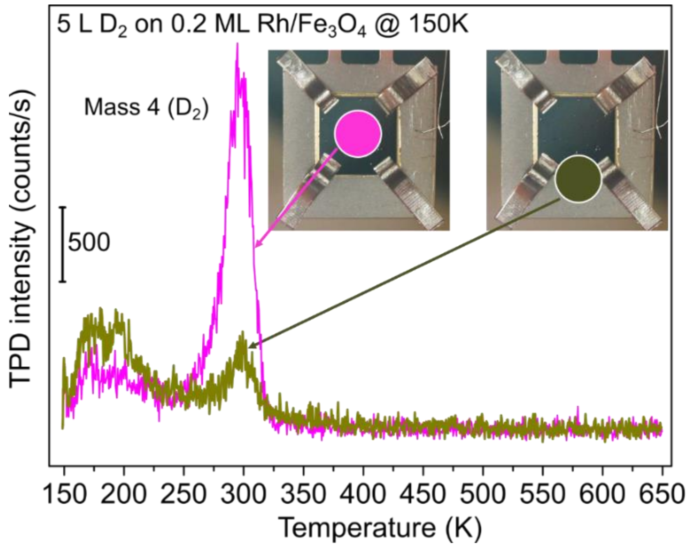

Figure S5. Control experiment to assess D_2 adsorption on the Ta sample plate. TPD spectra following 5 L D_2 exposure at 150 K on 0.2 ML Rh/Fe_3O_4(001). In the standard configuration (pink), D_2 is delivered via the molecular beam onto a 3.5 mm diameter area centered on the square sample. In the control experiment (olive green), the sample was displaced by 3.5 mm so that D_2 was directed primarily onto the Ta sample plate, with only a small contribution from on Rh/Fe_3O_4(001). The low-temperature

desorption peak is stronger in the control spectrum, suggesting it originates from D_2 adsorbed on the Ta plate, not on the $Fe_3O_4(001)$ surface.

Figures S4–S5 investigate the origin of the low-temperature shoulder or peak (marked by a black arrow in Figure S4) at 150−220 K observed in the D_2 TPD spectra. Initially, we hypothesized that this feature might arise from D_2 adsorption on a minority population of fivefold coordinated Rh adatoms (Figure S1b, green arrow) formed during deposition.[15b] To test this, we prepared a surface enriched in fivefold Rh sites by annealing a 0.2 ML twofold Rh/$Fe_3O_4(001)$ sample to 420 K, a treatment known to promote conversion from twofold Rh adatoms to embedded species, thereafter only a few twofold Rh sites remain.[17] As shown in Figure S4, this treatment does not increase the intensity of the shoulder peak, suggesting that fivefold Rh is not its origin.

We next considered whether the feature could arise from the Ta sample plate. Given the low sticking coefficient of D_2 on 0.2 ML Rh/$Fe_3O_4(001)$ surface at 200 K, most of the dosed molecules are expected to scatter and be pumped away, but some may be scattered and adsorb on the surrounding sample mount. To test this, we intentionally displaced the sample by 3.5 mm so the molecular beam predominantly targeted the Ta plate. The resulting TPD trace (Figure S5, olive green) shows a pronounced low-temperature peak, suggesting that the feature originates from D_2 adsorption on the Ta substrate, not from Rh on $Fe_3O_4(001)$.

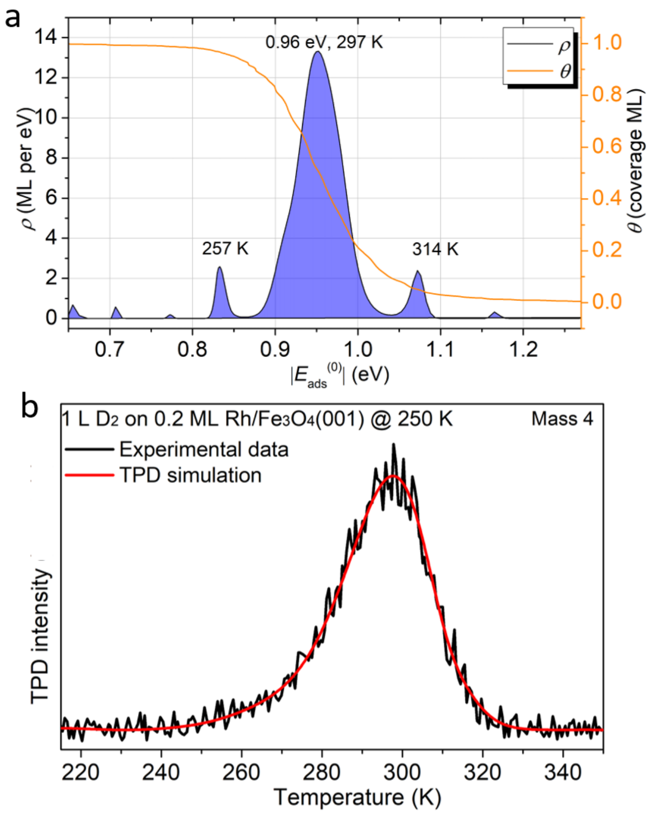

Figure S6. Extraction and validation of adsorption energy from D_2 TPD data. (a) Adsorption energy distribution $\rho(E_{ads}^{(0)})$ extracted from the experimental TPD spectrum following 1 L D_2 exposure on 0.2 ML Rh/Fe_3O_4(001) at 250 K, using a recently developed TPD analysis framework.[18] The model assumes adsorption of D_2 on twofold coordinated Rh_1 sites. For the analysis, the density of adsorption sites (defined as 1 ML in this plot) was taken as the nominal Rh coverage, 2.82×10^{13} cm^{-2}, the sticking probability was assumed as Langmuirian sticking with a zero-coverage sticking coefficient of 0.05, corresponding to a capture area of 18 Å^2 per unoccupied Rh atom (a rough guess). The analysis also depends on low-frequency vibrational modes, which contribute both to the energy and (more importantly) entropy. The DFT-calculated values are 10, 13 and 38 meV for Rh without an adsorbate, 14, 16, and 34 meV for Rh with two D atoms (dihydride), and the vibrational modes of the two adsorbed D atoms are at 39, 60, 63, 64, 193, 196 meV. The gas-phase D–D stretch is frozen at the relevant temperature and can be neglected. Based on this analysis, the main desorption peak at 297 K corresponds to an adsorption energy of 0.96 eV. The small side peaks in (a) are caused by noise. Assuming an error by a factor of 10 in the sticking yields an adsorption energy errors of 0.06 eV. The errors induced by the uncertainty of the vibrational energies are negligible (5 meV for a change by 30%). (b) Simulated TPD trace generated from the extracted energy distribution (red curves) overlaid on the experimental spectrum (black curve), showing good agreement between model and experiment.

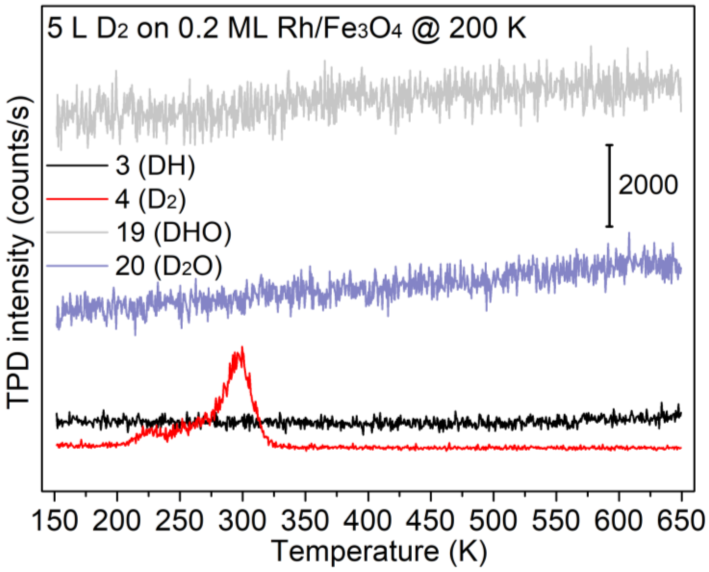

Figure S7. TPD spectra with a high dose. TPD spectra recorded following exposure of 0.2 ML Rh/Fe_3O_4(001) to 5 L D_2 at 200 K. This experiment was designed to test whether increased D_2 exposure promotes hydrogen spillover from isolated Rh sites to the Fe_3O_4 support, potentially leading to hydroxyl formation and subsequent water desorption during TPD. As in the 1 L D_2 experiment (Figure 3b), no water desorption is observed, indicating that spillover and formation of stable OD species do not occur even under extended D_2 exposure.

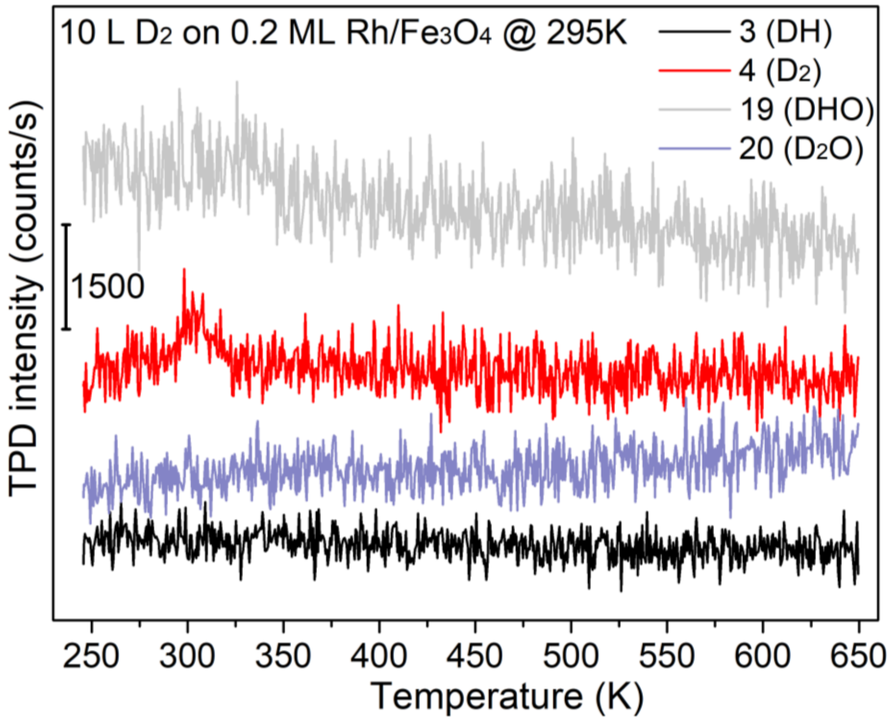

Figure S8. TPD spectra following high-dose D_2 exposure near the desorption temperature. A 0.2 ML Rh/Fe_3O_4(001) sample was exposed to 10 L D_2 at 295 K, cooled to 250 K, and then subjected to TPD measurements from 250–650 K, monitoring masses 3, 4, 19, and 20. Only a weak D_2 desorption signal was detected near 300 K, indicating negligible adsorption at 295 K. The absence of water desorption further confirms that no D_2 dissociation or spillover occurs under these conditions.

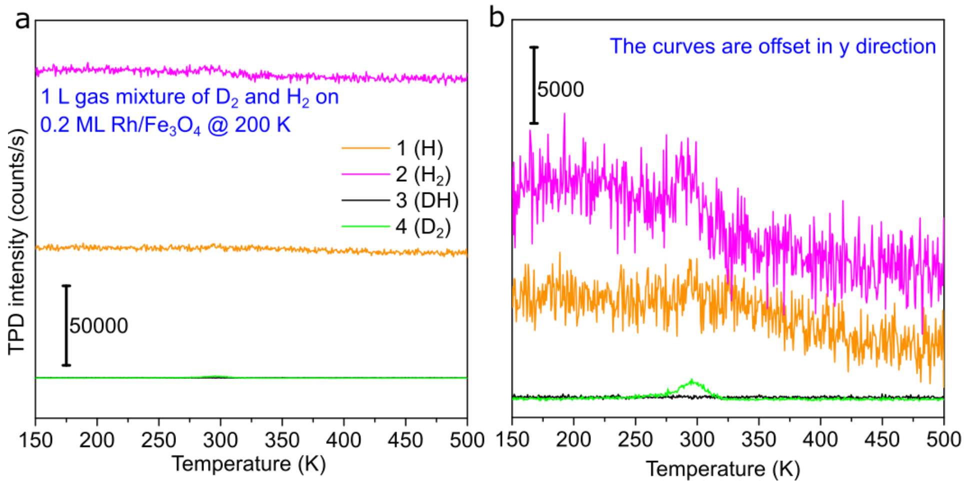

Figure S9. H–D exchange experiment to test for hydrogen spillover. A 1:1 mixture of H_2 and D_2 was dosed onto a 0.2 ML Rh/Fe_3O_4(001) sample at 200 K. TPD spectra monitoring masses 1, 2, 3, and 4 are shown in (a), with curves offset in (b) for clarity. Despite the presence of both H_2 and D_2 adsorption signals, no DH signal (mass 3) was detected. The high background of H_2 in UHV limits the signal-to-noise ratio for mass 1 and 2, which is why D_2 is preferred in surface science studies of hydrogen activation. This experiment is a test for the scenario of hydrogen spillover to the support, but desorption not in the form of water (which has been excluded in figures S7, S8) but via reverse spillover to the Rh, where it would recombine and desorb. In this scenario, isotopic scrambling, i.e., the formation of DH should be possible: H from H_2 adsorption at one Rh and D from D_2 adsorbed at a nearby Rh may desorb as DH; alternatively, DH could be formed by H and D from sequential adsorption events at the same Rh (each followed by spillover). The absence of DH (black trace) supports the conclusion that hydrogen activation is localized at Rh_1 sites without spillover onto the Fe_3O_4 support.

Table S1. DFT-calculated adsorption energies for dihydride and dihydrogen species on 2-fold coordinated $Rh_1/Fe_3O_4(001)$ using three different functionals: PBE+D3(BJ), r^2SCAN, and HSE06, as well as an RPA calculation to better account for electronic correlation. RPA and HSE06 energies were corrected for zero-point vibrational contributions obtained from HSE06 frequencies. The cell size was $(2\sqrt{2} \times 2\sqrt{2})R45°$ for PBE+D3(BJ) and r^2SCAN. For the computationally more demanding HSE06 and RPA calculations, a $(\sqrt{2} \times \sqrt{2})R45°$ cell was used. The E_{ads} difference for $2H/Rh_1$ (dihydride) between these cell sizes is ≈0.06 eV at PBE+D3(BJ) level.

	PBE + D3(BJ)		r^2SCAN		HSE06		RPA	
	ΔE_{ads} (eV)	$\Delta E_{ads,(ZPE)}$ (eV)	ΔE_{ads} (eV)	$\Delta E_{ads,(ZPE)}$ (eV)	ΔE_{ads} (eV)	$\Delta E_{ads,(ZPE)}$ (eV)	ΔE_{ads} (eV)	$\Delta E_{ads,(ZPE)}$ (eV)
$2H/Rh_1$ Dihydride	−1.31	−1.11	−1.04	−0.86	−1.47	−1.28	−1.33	−1.14
H_2/Rh_1 Dihydrogen	−1.08	−0.89	−0.98	−0.81	−1.14	−0.97	−1.15	−0.98
$2D/Rh_1$ Dihydride	−1.31	−1.16	−1.04	−0.90	−1.47	−1.33	−1.33	−1.19
D_2/Rh_1 Dihydrogen	−1.08	−0.94	−0.98	−0.85	−1.14	−1.02	−1.15	−1.03

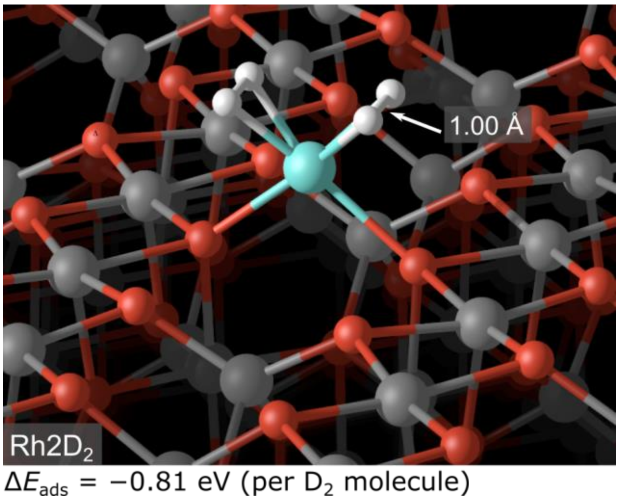

Figure S10. Simultaneous adsorption of two D_2 molecules on 2-fold coordinated Rh_1/Fe_3O_4 (001). DFT calculations show that two D_2 molecules can coordinate to a single Rh_1 site in the form of dihydrogen, with a total adsorption energy of −1.62 eV (PBE+D3(BJ)).

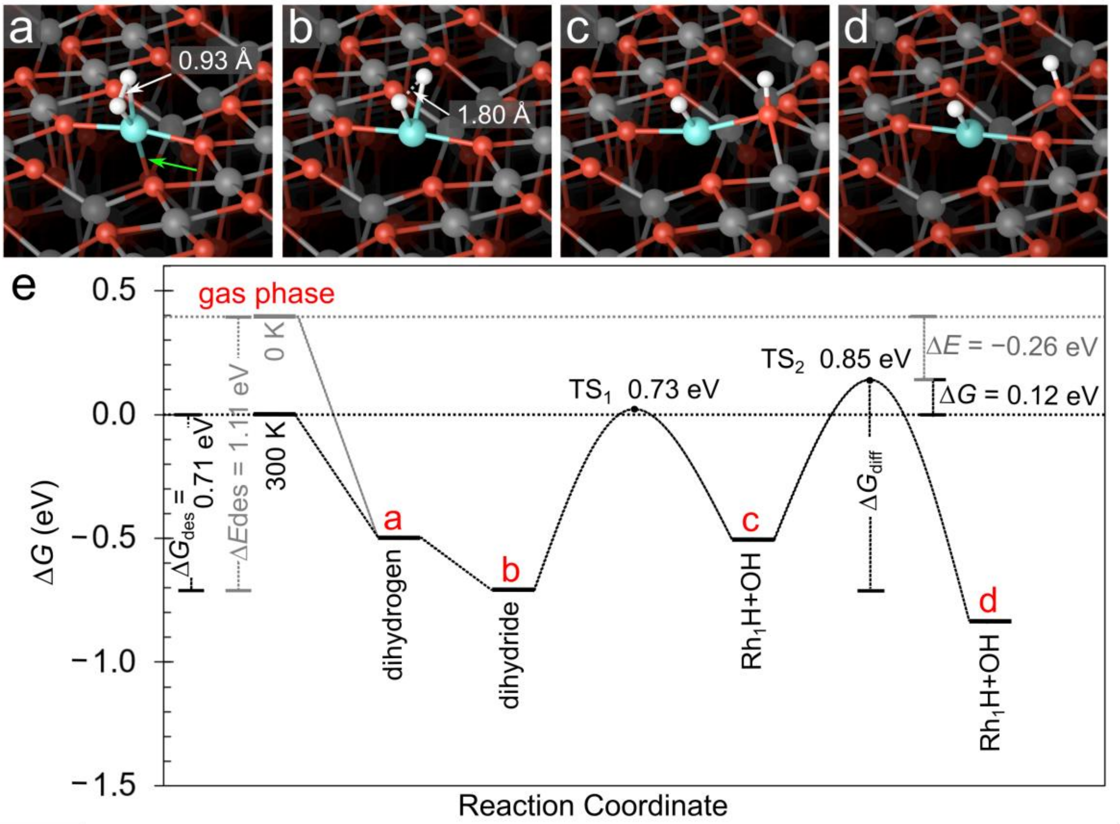

Figure S11. DFT-calculated mechanism of H_2 dissociation and spillover on $Rh_1/Fe_3O_4(001)$. Perspective views are shown for (a) a dihydrogen configuration and (b) a dihydride configuration. In both cases, the Rh_1 species is bond to two surface oxygen atoms. An additional weak bond from the Rh to a subsurface O atom (roughly 0.3 Å shorter than in the dihydride configuration) is indicated by the green arrow. (c-d) Atomic structures of atomic H spillover along the $Fe_3O_4(001)$ surface. Fe atoms are shown in dark gray, and oxygen atoms are red. (e) Computed reaction energy profile for molecular H_2 adsorption, dissociation, and hydrogen spillover from a Rh_1 site onto the $Fe_3O_4(001)$ substrate. The reported Gibbs free energy (ΔG) values incorporate the entropy difference between the adsorbed state ($S_{ads} = 0$) and the gas phase (S_{gas}) (as referenced in database).[8a] These ΔG values are presented for the experimental desorption temperature (300 K) and derived from DFT calculations that include ZPE corrections. The overall reaction mechanism remains identical to the 0 K pathway, except the desorption step, for which the 0 K desorption energy (ΔE_{ads}) is provided for comparative reference. Transition states (TS_1 and TS_2) and the corresponding activation barriers with respect to the dihydride state (b) are indicated.

Table S2. DFT-calculated vibration frequencies of dihydride adsorption on Rh/Fe_3O_4(001). The first two values are Rh–H(D) stretch, the next four are H(D) wagging, and the last three are Rh modes. Rh frequencies before adsorption are: 37.7 meV, 12.8 meV, and 10.0 meV.

2H/Rh/Fe_3O_4(001)	275.8 meV, 272.6 meV, 89.5 meV, 88.4 meV, 83.8 meV, 53.1 meV, 34.5 meV, 16.6 meV, 14.5 meV
2D/Rh/Fe_3O_4(001)	196.1 meV, 193.4 meV, 64.3 meV, 62.7 meV, 59.6 meV, 38.5 meV, 33.6 meV, 16.4 meV, 14.2 meV

References

[1] R. Bliem, E. McDermott, P. Ferstl, M. Setvin, O. Gamba, J. Pavelec, M. Schneider, M. Schmid, U. Diebold, P. Blaha, *Science* **2014**, *346*, 1215-1218.

[2] J. Pavelec, J. Hulva, D. Halwidl, R. Bliem, O. Gamba, Z. Jakub, F. Brunbauer, M. Schmid, U. Diebold, G. S. Parkinson, *The Journal of Chemical Physics* **2017**, *146*, 014701.

[3] G. Kresse, J. Furthmüller, *Computational Materials Science* **1996**, *6*, 15-50.

[4] a) G. Kresse, D. Joubert, *Physical Review b* **1999**, *59*, 1758; b) P. E. Blöchl, *Physical Review B* **1994**, *50*, 17953.

[5] J. P. Perdew, K. Burke, M. Ernzerhof, *Physical Review Letters* **1996**, *77*, 3865.

[6] S. Grimme, S. Ehrlich, L. Goerigk, *Journal of Computational Chemistry* **2011**, *32*, 1456-1465.

[7] a) A. Kiejna, T. Ossowski, T. Pabisiak, *Physical Review B* **2012**, *85*, 125414; b) I. Bernal-Villamil, S. Gallego, *Journal of Physics: Condensed Matter* **2014**, *27*, 012001.

[8] a) M. W. Chase, *Journal of Physical and Chemical Reference Data* **1998**, *28*, 1951; b) H. Falsig, B. Hvolbæk, I. S. Kristensen, T. Jiang, T. Bligaard, C. H. Christensen, J. K. Norskov, Angewandte Chemie International Edition **2008**, *47*, 4835.

[9] G. Henkelman, H. Jónsson, *The Journal of Chemical Physics* **2000**, *113*, 9978-9985.

[10] J. W. Furness, A. D. Kaplan, J. Ning, J. P. Perdew, J. Sun, *The Journal of Physical Chemistry Letters* **2020**, *11*, 8208-8215.

[11] G. Sai Gautam, E. A. Carter, *Physical Review Materials* **2018**, *2*, 095401.

[12] A. V. Krukau, O. A. Vydrov, A. F. Izmaylov, G. E. Scuseria, *The Journal of Chemical Physics* **2006**, *125*.

[13] M. Kaltak, J. Klimeš, G. Kresse, *Physical Review B* **2014**, *90*, 054115.

[14] O. Gamba, J. Hulva, J. Pavelec, R. Bliem, M. Schmid, U. Diebold, G. S. Parkinson, *Topics in Catalysis* **2017**, *60*, 420-430.

[15] a) C. Wang, P. Sombut, L. Puntscher, Z. Jakub, M. Meier, J. Pavelec, R. Bliem, M. Schmid, U. Diebold, C. Franchini, *Angewandte Chemie International Edition* **2024**, 63, e202317347; b) Z. Jakub, J. Hulva, P. T. Ryan, D. A. Duncan, D. J. Payne, R. Bliem, M. Ulreich, P. Hofegger, F. Kraushofer, M. Meier, *Nanoscale* **2020**, *12*, 5866-5875.

[16] a) M. Meier, J. Hulva, Z. Jakub, J. Pavelec, M. Setvin, R. Bliem, M. Schmid, U. Diebold, C. Franchini, G. S. Parkinson, *Proceedings of the National Academy of Sciences* **2018**, *115*, E5642-E5650; b) T. Kendelewicz, S. Kaya, J. Newberg, H. Bluhm, N. Mulakaluri, W. Moritz, M. Scheffler, A. Nilsson, R. Pentcheva, G. Brown Jr, *The Journal of Physical Chemistry C* **2013**, *117*, 2719-2733.

[17] C. Wang, P. Sombut, L. Puntscher, M. Ulreich, J. Pavelec, D. Rath, J. Balajka, M. Meier, M. Schmid, U. Diebold, *The Journal of Physical Chemistry C* **2024**, *128*, 15404-15411.

[18] M. Schmid, G. S. Parkinson, U. Diebold, *ACS Physical Chemistry Au* **2022**, *3*, 44-62.
